# Effects of Biological Therapies on Molecular Features of Rheumatoid Arthritis

**DOI:** 10.3390/ijms21239067

**Published:** 2020-11-28

**Authors:** Chary Lopez-Pedrera, Nuria Barbarroja, Alejandra M. Patiño-Trives, Maria Luque-Tévar, Eduardo Collantes-Estevez, Alejandro Escudero-Contreras, Carlos Pérez-Sánchez

**Affiliations:** Rheumatology Service, Reina Sofia Hospital, Maimonides Institute for Research in Biomedicine of Cordoba (IMIBIC), University of Cordoba, E-14004 Córdoba, Spain; nuria.barbarroja.exts@juntadeandalucia.es (N.B.); alejandramaria.patino@gmail.com (A.M.P.-T.); merluqtev@gmail.com (M.L.-T.); educollantes@yahoo.es (E.C.-E.); alexcudero@hotmail.com (A.E.-C.); b32pesac@uco.es (C.P.-S.)

**Keywords:** rheumatoid arthritis, autoimmunity, inflammation, oxidative stress, NETosis, genome, epigenetic and pos-transcriptional mechanisms, bDMARDs

## Abstract

Rheumatoid arthritis (RA) is an autoimmune and chronic inflammatory disease primarily affecting the joints, and closely related to specific autoantibodies that mostly target modified self-epitopes. Relevant findings in the field of RA pathogenesis have been described. In particular, new insights come from studies on synovial fibroblasts and cells belonging to the innate and adaptive immune system, which documented the aberrant production of inflammatory mediators, oxidative stress and NETosis, along with relevant alterations of the genome and on the regulatory epigenetic mechanisms. In recent years, the advances in the understanding of RA pathogenesis by identifying key cells and cytokines allowed the development of new targeted disease-modifying antirheumatic drugs (DMARDs). These drugs considerably improved treatment outcomes for the majority of patients. Moreover, numerous studies demonstrated that the pharmacological therapy with biologic DMARDs (bDMARDs) promotes, in parallel to their clinical efficacy, significant improvement in all these altered molecular mechanisms. Thus, continuous updating of the knowledge of molecular processes associated with the pathogenesis of RA, and on the specific effects of bDMARDs in the correction of their dysregulation, are essential in the early and correct approach to the treatment of this complex autoimmune disorder. The present review details basic mechanisms related to the physiopathology of RA, along with the core mechanisms of response to bDMARDs.

## 1. Introduction

Rheumatoid arthritis (RA) is a systemic inflammatory autoimmune disease identified by continuous joint inflammation promoting cartilage and bone damage, incapacity, and eventually systemic complications. The progression of the disease promotes loss of function, reduced quality of life and increased morbidity and mortality. In addition, RA can also affect organs outside the joints, mainly the lungs, skin, and cardiovascular system.

The precise etiology of RA is still unknown, but genetic and environmental stimuli clearly participate. Dysregulated adaptive immunity can precede the clinical manifestations for many years, and it is likely that the repeated activation of innate immunity can contribute to a breakdown of tolerance.

Regarding the mechanism of RA, it was shown that post-translational modifications in the mucosa (such as citrullination or carbamylation) generate new epitopes recognized by the adaptive immune system. Modified peptides are then presented by APC (antigen-presenting cells) that in turn activate in lymphoid tissues the adaptive immune response and promote autoantibodies formation. Fibroblast-like synovial cells (FLS), macrophages and APCs can be activated in the synovium to produce several inflammatory factors. Next, the autoimmune response caused by the immune system generates synovial inflammation, thus promoting cytokine secretion and synovial blood vessel leakage. Lastly, the autocrine and paracrine effects of cytokines and the continuous adaptive immune response can make the disease permanent and eventually lead to cartilage and bone destruction [1].

In addition, oral infections with specific microorganisms such as Porphyromonas gingivalis have been associated with the RA development. The pathogenic mechanisms involved in the relationship of periodontal disease and RA comprise the increased production of T helper 17 by the microorganisms, the burden of citrullination, the generation of self-antigens that may cross-react with host protein and convert to autoantigens and the possibility of the bacterial translocation to the joints increasing the inflammatory burden leading to the osteoclastic activation, among others. All these mechanisms can trigger the onset of RA and the perpetuation of the inflammation, worsening the progression of the disease (reviewed in Rooney C.M. et al., 2020) [2].

**Figure 1:**
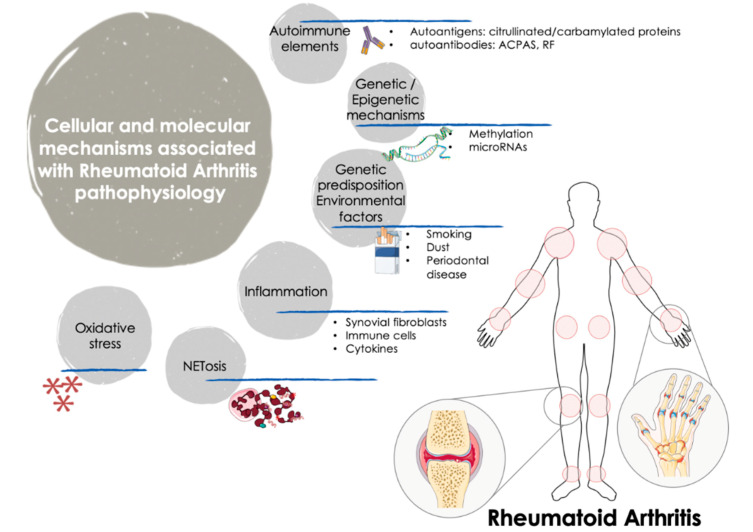
Cellular and molecular mechanisms involved in the pathogenesis of RA. The onset and progression of RA is orchestrated by several cellular and molecular pathologic mechanisms that participate in an integrated and coordinated way. The interaction between multiple genetic and environmental factors increase the risk of RA development. Autoimmune factors such as autoantigens and autoantibodies along with inflammatory mediators released by activated immune cells -including several cytokines and chemokines- are well recognized molecular features that influence the clinical manifestations of RA, characterized by tender and swollen joints and other related comorbidities. In recent years, novel mechanisms have been further associated with the pathogenesis of RA, including oxidative stress and NETosis and the interplay among gene expression and epigenetic and post-transcriptional mechanisms such as methylation and microRNAs. ACPAS, Anti-citrullinated peptide antibodies; RF, Rheumatoid Factor.

RA is a dissimilar disease, involving multiple clinical manifestations and pathogenic mechanisms among individuals with the same diagnosis and/or throughout different disease stages. Indeed, although autoantibodies are an important feature of RA, several RA patients are negative for these autoantibodies. These traits further support the complexity of the disease and the involvement of numerous factors in the trigger and the evolution of RA (Figure 1).

Thus, it was widely demonstrated that along with synovium and immune cells, cytokines play key roles in the pathophysiology of the disease, through their involvement in cell growth, proliferation, differentiation, tissue repair and regulation of the immune response, being responsible for inflammation and joint destruction, and even for the systemic involvement in this autoimmune disorder [3].

RA is also related to oxidative stress. In RA patients, reactive oxygen species (ROS) produced by joints and circulating neutrophils, monocytes and macrophages can damage various cell constituents, including carbohydrates, proteins, lipids, and DNA. Accordingly, ROS may induce death of chondrocytes and promote a higher inflammatory damage both at articular and systemic levels. Moreover, a robust positive correlation between free radicals (generated both in synovial fluid and peripheral blood) and DAS28 score was proven, underscoring that ROS can be considered a predictive marker for the inflammatory status in patients with RA [4].

Accelerated NETosis (the process wherein neutrophils release highly decondensed chromatin and granular contents to the extracellular space, called neutrophil extracellular traps -NETs-) was also related to the pathogenesis of RA patients, through the externalization of immunostimulatory molecules and citrullinated autoantigens that produce abnormal adaptive and innate immune responses both in the joint and in the periphery, thus perpetuating damaging mechanisms and fostering the development of related comorbidities in this disease [5].

From the genetic standpoint, recent studies (including both gene-arrays and new generation sequencing studies) uncovered the gene expression signatures and networks in synovial tissue and purified immune cells associated with the pathogenesis of RA [6]. Thereafter, epigenetic mechanisms underlying gene regulation, and documented to be altered in RA were also evaluated. Thus, genome-wide methylation studies identified several changes in FLS and immune cells linked to the origin and the development of this disorder [7]. In addition, the study of post-transcriptional mechanisms of gene regulation identified several microRNAs directly associated with the disease activity, the production of inflammatory mediators and the osteoblastogenesis [8].

The unmet need for new treatment alternatives for RA patients encouraged research in the development of novel drugs regulating physiologically relevant targets. In this context, biologic immunosuppressive therapies (named biologic disease-modifying antirheumatic drugs, bDMARDs) have proven to control disease activity and halt the progressive joint destruction. These drugs were designed to target the inflammatory molecules, cells, and pathways that confer tissue damage in RA patients.

This review is focused on the latest insights related to the potential role of bDMARDs in the regulation of the most significant processes that control the pathogenesis of RA, through the analysis of their involvement in the control of inflammation, oxidative stress, and NETosis, and the underlying transcriptomic, epigenetic, and post-transcriptional mechanisms.

## 2. Autoimmunity and Inflammation

### 2.1. The Fundamental Role of Autoantibodies, Fibroblasts, Immune Cells and Cytokines in the Progression of RA

Several autoimmune and cellular constituents can be pondered as key factors for the occurrence and development of RA, including autoantibodies, synovial fibroblasts, and white blood cells such as monocytes, lymphocytes, and neutrophils. The activation of these cells promotes the production of inflammatory mediators and cytokines, thereby perpetuating this pathogenic process.

Most patients with RA will develop anti-citrullinated protein antibodies (ACPAs). Therefore, in the early stages of RA development, tolerance to citrullinated antigens is disturbed, and the anti-citrullinated humoral response was expanded, fostering the development of inflammation and eventually arthritis.

The biological activities of ACPAs include stimulating the production of pro-inflammatory cytokines, inducing osteoclast formation, and promoting the release of autoantigens by neutrophils [9]. As such, in a previous study, we demonstrated that ACPAs purified from RA patients fostered the induction of pro-oxidation, pro-inflammatory and atherosclerotic states of different white blood cell subpopulations [10]. In addition, an effect on macrophage activation and survival was also confirmed. Therefore, ACPA-mediated processes might contribute to the progress and/or prolongation of RA.

FLS are key components of RA infiltrating synovium and play a major role in the occurrence and persistence of destructive joint inflammation. The pathogenic capacity of FLS in RA derives from its capability to secrete immunomodulatory cytokines and inflammatory mediators, as well as multiple matrix modeling enzymes and adhesion molecules [11].

Likewise, monocytes/macrophages play a major role in inflammation by releasing cytokines and infiltrating extensively at sites of inflammation, such as synovium. In addition, given that monocytes/macrophages are ‘over-differentiated’ into osteoclasts, they are linked to bone erosion in RA. Besides, the increase in the number of macrophages in the lower layer of the synovium is an early feature of active rheumatism, so that the presence of high number of macrophages is the main feature of inflammatory lesions. Moreover, the degree of infiltration of synovial macrophage is associated with the degree of joint erosion, and the removal of these macrophages from inflamed tissues has great therapeutic benefits [12,13,14].

Lymphocytes are the most studied cells that mediate the pathogenesis of RA, so that RA has been largely considered to be a disease driven by T helper (Th) 1 cells. Th1/Th2 is imbalanced, being higher the levels of Th1 cells. Th1 secretes inflammatory molecules, including interleukin (IL)-2, interferon-γ (IFNγ), and tumor necrosis factor-α (TNFα), thus preventing the differentiation of CD4^+^ T cells into Th2 cells [15]. Th17 lymphocytes were also associated with the pathogenesis of RA, mainly producing IL-17A, IL-6 and TNFα. These cytokines are expanded in RA patients’ serum and promote the activation of other cells.

Regulatory T cells (Treg) are also involved in the RA pathology. They play an important role in immunosuppression through cell-cell interactions, and are significantly reduced in the late stage, and show impaired function in RA. Therefore, it was proposed that the development and progression of RA are caused by the imbalance of Th1/Th2 and Th17/Treg cells [15,16].

Equally, B lymphocytes play a fundamental role in the pathogenesis of RA. They are a source of ACPAs and RF, and they contribute to the formation of immune complexes and the stimulation of complement in joints. B cells are also very effective antigen-presenting cells and stimulate the activation of T cells by co-stimulatory molecules. In addition, B cells both produce and respond to chemokines and cytokines, thereby promoting the infiltration of leukocytes into joints, the ectopic lymphoid structures formation, angiogenesis and synovial hyperplasia [3].

Finally, among cells of the immune compartment, neutrophils are the most abundant cells in the synovial fluid of the damaged joints and in the pannus/cartilage interface where tissue injury occurs. The pathogenic role of neutrophils in RA involve changes in several processes, including augmented cell survival and migration, abnormal inflammatory activity, increased oxidative stress and release of NETs. By activating these mechanisms, neutrophils can stimulate other immune cells, thereby perpetuating inflammation and promoting the destruction of injured articular cartilage and bones [17].

Cytokines, together with synovial cells and immune cells, and as products and controllers of the immune system in RA, play vital biological processes, such as cell growth, differentiation, proliferation, inflammation, tissue repair and immune response regulation. This causes inflammation and joint destruction that occurs during arthritis development [18].

Hence, the characteristic inflammation of RA is due to the predominance of pro-inflammatory cytokines over those of anti-inflammatory cytokines.

A myriad of studies supports that the most relevant pro-inflammatory cytokines in the pathogenesis of RA are TNFα, IL-1, -6, -7, -12, -17, -18, -23, and -32. They are mostly produced by myeloid cells, fibroblasts, endothelial cells, keratinocytes and chondrocytes, which are also involved in the production of metalloproteinases, oxidative stress mediators and prostaglandins that lead to inflammation, osteoclast activation and differentiation, osteoclastogenesis, neovascularization and angiogenesis [19].

Instead, several anti-inflammatory cytokines counterbalance the chronic activation of RA immune cells. Thus, Th2 cells and a set of innate lymphoid cells (ILC2s) secrete IL-4 and IL-13. These cytokines induce in macrophages the differentiation into a regulatory M2 phenotype via signal transducer and activator of transcription 6 (STAT6) activation. Then, M2 macrophages release transforming growth factor beta (TGFβ) and anti-inflammatory cytokines such as IL-10. Moreover, IL-5 produced by ILC2s and Th2 cells recruit eosinophils, which shift the balance of macrophages to primarily being regulatory M2 macrophages, thus diminishing the secretion of pro-inflammatory cytokines such as IL1β and TNFα by M1 macrophages [20].

In this complex landscape, it is further proven that leukocyte recruitment is a key process in regulating the onset of inflammation [21]. Throughout the course of life, a subset of white blood cells continues to enter, pass through, and leave the matrix of the bone marrow, thymus, lymph nodes and surrounding tissues.

Patients with RA have defects in several checkpoints regulating leukocyte entry and exit from lymphoid tissues and the joints.

Previous studies have shown that synovial endothelial cells (first barrier that leukocytes must traverse to enter the joint) from RA patients induce the recruitment of leukocytes after TNFα stimulation [22]. Actually, the rheumatoid joint microenvironment was demonstrated to promote the phenotypical alteration of synovial endothelial cells [23]; such cells have increased expression of the adhesion molecules vascular adhesion molecule 1 (VCAM1) and E-selectin together with enlarged expression of both peripheral node addressin (PNAd) and vascular adhesion protein 1 (VAP1) [24]. Thus, leukocyte entry into the join depends on the phenotype of its gatekeepers, the synovial endothelial cells.

Another factor to consider in this tightly regulated process is the self-regulation of leukocyte trafficking. Thus, it was shown that a B cell derived peptide termed PEPITEM, a key constituent of the immunoprotective pathway that reduces the T cell trafficking into inflamed tissues, is absent in patients with RA [25]. Hence, in RA patients, circulating B cells express reduced levels of receptors for adiponectin, do not secrete PEPITEM in response to adiponectin and are thus unable to suppress T cell migration in vitro.

That overall data point at a significant heterogeneity in leukocyte trafficking patterns in RA patients, which are further involved in both the evolvement of the disease and the response to several therapies [26].

### 2.2. Biological DMARDs as Effective Therapies to Reduce Inflammation and Articular Damage

The latest type of treatments used for RA therapy is biological compounds or biological response modifiers, designed to target inflammatory molecules, cells, and pathways that cause tissue damage in RA patients.

**Figure 2:**
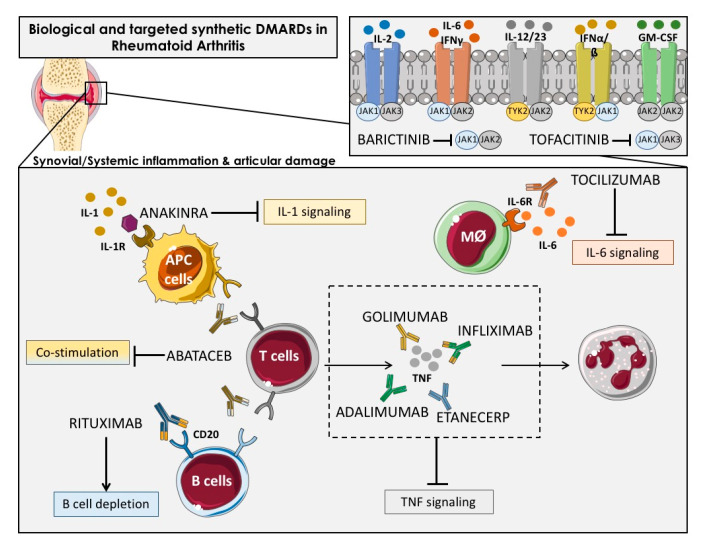
Overview of the main immunologic pathways targeted by the biological and targeted synthetic therapies in rheumatoid arthritis. Simplified description of cytokines and receptors that are targeted by available biologic DMARDs such as: TNF inhibitors (adalimumab, golimumab, infliximab and etanercept), IL-1R antagonist (anakinra), antibody against IL-6R (tocilizumab) and anti-CD20 (rituximab), and targeted synthetic DMARDs, such as Janus kinase inhibitors (anti-JAKs): tofacitinib (JAK1-JAK3 inhibitor) and baricitinib (JAK1–JAK2 inhibitor). APC: Antigen-presenting cells; Mφ: Macrophage.

The bDMARDs currently approved and clinically demonstrated as most effective include TNFα inhibitors (infliximab, etanercept, adalimumab, etc.), T cell costimulation modulators (abatacept), IL-6-inhibitors (tocilizumab, sarilumab), IL-1 inhibitors (anakinra), and anti-CD20 drugs (rituximab). Most of them are direct inhibitors of pro-inflammatory cytokines, while others develop their function upstream of the inflammatory cascade, thereby interfering the activation of T cells (i.e., abatacept) or the depletion of B cells (i.e., ituximab) [27] (Figure 2).

New encouraging treatments for RA patients encompassed the inhibition of various cytokines by the inhibitors of the Janus kinase family (JAK) [28] including tofacitinib, the first JAK-inhibitor agreed for RA treatment, which inhibits JAK-3 and -1, and baricitinib, which is selective for JAK-1 and -2 [29]. JAK inhibitors, particularly JAK1 inhibitors, can prevent activation of transcription factors of Signal Transducer and Activator of Transcription STAT transcription factors in the synovium and are effective RA treatment drugs. Synovial biopsy studies have shown that the JAK-inhibitor tofacitinib is associated with clinical improvement of RA [30].

Numerous studies, including ours, demonstrated the anti-inflammatory effects of bDMARDs, both in vivo and in vitro, in RA patients [10,31,32].

Thus, several studies showed the capacity of biological therapies to reduce the protein levels of different cytokines and adhesion molecules. Bulur A. et al. reported that after 6 months of treatment with anti-TNF therapy including infliximab, adalimumab and etanercept, the serum levels of IL10, IL-12p40, IFN- and macrophage chemoattractant protein-1 (MCP-1) were reduced [33]. In line with this, Charles P. et al. demonstrated that the treatment with infliximab decreased the serum levels of IL-6, IL-1, GM-CSF, IL-8, p55 sTNFR in 73 RA patients after four weeks of treatment [34]. The reduction of IL-6 after anti-TNF therapy was validated by other studies since its activity is directly associated with TNF-alpha [35,36]. In addition to the decrease of IL-6, our group further demonstrated a reduction of IL17 and TNF-a in 96 RA patients treated with anti-TNF therapy for six months [36]. Other cytokines were diminished after TNF inhibitors in the synovium, including IL-8, MCP-1 [37], and IL1 [38]. We also showed that other biological therapies such as Tocilizumab was able to reduce the plasmatic levels of migration/adhesion molecules such as soluble VCAM and E-Selectin [39]. VEGF-A is another key inflammatory and vascular mediator that was shown to be reduced after tocilizumab treatment in RA patients [40].

Yet, recent reports demonstrated that bDMARD not only exert anti-inflammatory effects, but are involved in several mechanisms underlying the pathogenesis of RA. Thus, it was shown that anti-cytokine therapies systematically target many of the molecules involved in the leukocyte recruitment cascade; for example, TNFα and IL-1β are involved in endothelial cell activation and IL-6 is involved in fibroblast–endothelial cell communication. Adalimumab and adalimumab reduced granulocytes influx into the joints of RA-established patients. Treatment with IFX for two weeks also reduced articular T cell, B cell and macrophage counts in comparison to cell counts in pretreatment synovial tissue samples obtained by biopsy [37,41].

By contrast, treatment with adalimumab did not alter monocyte entry into the joints of patients with RA. Instead, adalimumab was suggested to reduce macrophage and monocyte numbers within the joint by influencing their egress [42]. Unlike etanercept both infliximab and adalimumab reportedly restored the function of peripheral blood Treg cells and increased the number of these cells in the circulation [43,44].

Other DMARDs that affect cytokine production, such as JAK inhibitors can also influence the migratory potential of leukocytes. Peripheral blood neutrophils from patients with RA migrate along an IL-8 chemotactic gradient in a JAK2-dependent manner; in vitro, this process was sensitive to the JAK1–JAK2 inhibitor baricitinib but not the JAK3–JAK1 inhibitor tofacitinib [45].

Up to date, there has been less success targeting many other signaling pathways, including p38 mitogen-activated protein kinase (MAPK), MAPK/ERK kinase, spleen tyrosine kinase and Bruton’s tyrosine kinase [46,47].

Several new therapeutic targets, including bDMARDs directed at the innate immunity, and the prevention of joint destruction and migration (inhibitors of IL-6, granulocyte macrophage colony-stimulating factor (GM-CSF), matrix metalloproteinases, chemokines) are now under study in different phases of clinical trials [48].

Overall, these data suggest that the multiple pathways involved in the clinical features of RA converge in different pathophysiological processes that may be simultaneously targeted by bDMARDs, thus offering many opportunities for therapeutic intervention.

## 3. Oxidative Stress

### 3.1. The Role of Oxidative Stress in the Physiopathology of Rheumatoid Arthritis

RA has been associated with oxidative stress, a pathologic status on which reactive oxygen species (ROS) increases over time, either by their increased production, the reduction in antioxidant defenses (mainly exemplified by enzymes such as catalase (CAT), superoxide dismutase (SOD), glutathione reductase (GR), glutathione peroxidase (GPx), and thioredoxin reductase), or through the arrangement of both eventually involving compromise in the redox signaling.

In RA patients, ROS produced in large quantities by joints and circulating neutrophils and monocytes/macrophages, can damage cell structures such as carbohydrates, DNA, proteins, and lipids [4,49]. Hence, clinical evidence indicates that patients with RA increased lipoperoxides (LPO), protein oxidation levels, and oxidative DNA damage.

Thus, several post-translational modifications of proteins occur under pro-oxidative conditions, which may generate neoepitopes acknowledged as ‘non-self’ by the immune system that lead to the formation of autoantibodies, thereby extending the pathologic process [50].

In this way, we previously demonstrated that ACPAs may act as direct inductors of a pro-oxidative status in monocytes and neutrophils, along with a pro-inflammatory profile in lymphocytes and monocytes [10].

While ROS production by phagocytes during the oxidative burst is essential to kill off pathogens, in the setting of inflammatory diseases, such as RA, phagocyte-derived ROS has also been connected with promotion of inflammation and tissue damage [51].

A positive feedback between oxidative stress and inflammation was further demonstrated in RA patients. Through these two interactions, the mutual destructive effect between both pathogenic processes is further heightened [4,52,53].

In this regard, pro-inflammatory cytokines are responsible for activating the MAPK, which in turn, promotes the activation of nuclear factor-κB (NF-κB). This molecule prompts the transcription of various genes involved in the maintenance of inflammation [54]. Moreover, NF-κB may, in addition to promote an increased secretion of IL-1 and TNFα, be activated by these cytokines, thereby generating a positive feedback during each other’s self-activation process [4].

Therefore, it was demonstrated that activated T cells and macrophages in the synovium induce the production of ROS through the release of TNFα and IL-1 [55], resulting in augmented synovitis [56].

Regarding the effects of oxidative stress on particular cellular types, ROS promote death of chondrocytes, contributing to the articular injury seen in early phases of RA [57]. Moreover, the immune dysregulation observed in autoreactive T lymphocytes may be associated with their exposure to an oxidative stress environment [58]. Furthermore, local macrophages may amplify the production of ROS by the proinflammatory intra-articular cascades [49] in conjunction with the local increased production of autoantibodies, given that both RF and ACPA are locally produced by B cells present in the synovium [59].

Thus, RA patients with active disease, display enlarged levels of ROS and reduced antioxidant potential, resulting in a poorer oxidative status and a greater inflammatory damage. Thus, it was described that serum LPO levels are positively correlated with pro-inflammatory cytokines in RA [60]. Besides, reactive oxygen metabolites, also increased in blood samples of RA patients, are positively correlated with disease activity [61]. Accordingly, lower levels of antioxidants were also found in the serum and the synovial fluid of RA patients [62].

All in all, it was reported that there is a strong positive correlation between free radicals (produced in peripheral blood or synovial fluid) and Disease activity score (DAS) 28 score, stressing that regardless of the source, the measurement of ROS, can be used as a predictor of the degree of inflammation in RA [63]. In addition, there is a close relationship between ACPA titers and oxidative stress, which supports the multiple pathogenic effects of ACPAs in RA.

### 3.2. Antioxidant Properties of Biologic DMARDs in Rheumatoid Arthritis

Numerous studies analyzing the effects of bDMARDs on oxidative and nitrosative stress in RA demonstrated the improvement in the redox state, either through the reduction in markers of oxidation or by the rinse in the antioxidant capacity [64].

TNF-α inhibitors (TNFi) exert an antioxidative stress activity interfering with the pleiotropic effect of this cytokine. TNF-α stimulates NAD(P)H oxidases activity and increases intracellular hydrogen peroxide. The up-regulation of TNF-α also impairs nitric oxide (NO) bioavailability and contributes to the development of mitochondrial oxidative stress [65].

In addition, the activation of the caspase cascade induced by TNF-α can promote endothelial cell apoptosis, accompanied by increased oxidative stress and vascular inflammation, and augments the risk of cardiovascular disease [66].

Many reports documented that TNFi (i.e., etanercept and infliximab) have positive effects on the oxidant damage of RA, promoting reduction in serum and urinary levels of oxidative DNA damage, myeloperoxidase activity, and lipid peroxidation, in parallel to an equivalent decrease in DAS28 [67].

Various studies also demonstrated an increase in antioxidant defenses, confirmed by increase in levels of thiols [68], SOD, CAT, GPx, and glutathione (GSH) [69,70].

Indeed, in RA patients responding to TNFα inhibitors, hypoxia levels in synovial tissue were found to be reduced, leading to a reduction in the hypoxic microenvironment, mitochondrial DNA mutations and a significant reduction in disease activity. Later on, it was shown that in RA patients who respond to TNFα blockade therapy, the level of the lipid peroxidation product 4-hydroxynonenal decreases, which corresponds to the significant increase in tissue oxygen. Consistently, in parallel with the decrease of hypoxia in the joint tissue the oxidative stress of the synovium is also reduced [71,72].

IL-6 is an inflammatory cytokine that plays a key role in propagating the inflammatory response, and further inducing atherosclerosis, because it is involved in endothelial dysfunction and arteriosclerosis, which helps an accelerated atherosclerotic process is observed in RA patients [73].

In addition, it plays a role in the differentiation of B lymphocytes into plasma cells producing autoantibodies, which contribute to the pathogenesis of RA by forming immune complexes [74].

Hence, IL-6 induces ROS production in RA synovial fibroblasts [75]. In this way, it was proved that serum oxidative stress levels were significantly reduced in RA patients receiving tocilizumab treatment [76]. Since then, another study confirmed that compared with anti-TNFα antibodies, serum levels of oxidative stress markers were significantly suppressed in RA patients treated with tocilizumab [77].

In a recent study, our research team further confirmed that subcutaneous TCZ for 6 months can restore endothelial function, inhibit the activity of monocytes and neutrophils, and reduce ROS and reactive nitrogen species in both cell types (peroxides and peroxynitrites) [39]. That overall data shows that tocilizumab has a robust effect in reducing oxidative stress in RA patients.

In RA patients, IL-1 increases IL-6 and C-reactive protein (CRP) and endothelin-1 production [78,79,80,81], and stimulates superoxide anion release [82].

IL-1 also causes an excess of NO- release [83] which generate peroxynitrites after reacting with the superoxide anion [84]. This molecule then nitrates tyrosine, leading to the generation of nitrotyrosine, a marker of protein nitrosative stress [85].

Indeed, in RA patients, the inhibition of IL-1 activity by treatment with anakinra (single injection 100 mg, subcutaneous) abridged the oxidative stress through the down-regulation of lypoperoxides, nitrotyrosine, and protein carbonyl content [86].

A precedent study demonstrated that IL-1 activity inhibition in RA patients by anakinra (subcutaneous single injection of 100 mg) abridged the oxidative stress through the down-regulation of protein carbonyl, nitrotyrosine, and lypoperoxides. In a similar study, single injection of anakinra (150mg SC) reduced nitrotyrosine, malondialdehyde (MDA), endothelin-1 and IL-6 levels, restoring the normal vascular and left ventricular function in RA patients [87].

RA patients responding to treatment with abatacept (a fusion protein that selectively inhibits T cell activation, T cell proliferation and B cell immunological response), showed a significant up-regulation of genes belonging to the electron transport chain pathway was observed (COX7B, COX11, NDUFU3, NDUFS1, UQCRC2), which are related to the restoration of the redox balance and the improvement of T cell response [88].

The presence of autoantibodies, and thus the role of B cells, is the basic feature of RA. In addition to the ‘vicious cycle’ of inflammation, one of the main characteristics of the disease is determined by the interaction between B cells and T cells. In fact, in addition to antibody-dependent mechanisms, B cells can also act as antigen-presenting cells and co-stimulate T lymphocytes and other immune cells, such as neutrophils and monocytes. This ultimately leads to an increase of oxidative stress, the activation and release of NETs, the secretion of cytokines and the over-expression of cell-surface ligands, which in turn activate other B cells.

Based on the evidence demonstrating the critical role of B cells in the pathophysiology of RA many studies evaluated the clinical impact of therapies that target B cells. Rituximab is a monoclonal antibody against CD20 molecules expressed on the surface of both pre-B and mature B lymphocytes. The mechanism of rituximab action comprises cell lysis by complement or antibody-dependent cell-mediated cytotoxicity, blocking of co-stimulatory molecules, apoptosis induction, and neutralization of auto-antibodies and inflammatory factors (cytokines and their receptors) [89].

Accordingly, in a recent study [90] we verified a reduction of the pro-oxidative status in RA patients after three months of rituximab therapy, as demonstrated for the down-regulation in the serum levels of LPO. That reduction is likely due to the parallel inhibition of rituximab-promoted autoantibody and pro-inflammatory molecules production.

In sum, the pharmacological therapy with biologic DMARDs promotes an improvement in the redox state of RA patients that is further related to the success of the therapy. Therefore, the measurement of oxidative stress-related biomolecules may be helpful to evaluate disease activity and therapy effectiveness.

## 4. NETosis in Rheumatoid Arthritis

### 4.1. Role of NETs on the Pathophysiology of RA and Its Comorbidities

Emerging evidence shows that neutrophils also play a very important role in the initiation and perpetuation of RA through direct effects on the synovium and the regulation of joint and systemic innate and adaptive immune responses [91]. In addition, recent work showed that neutrophils may promote the generation of modified self-antigens, further affect the interaction among antigen-specific T cells and autoantibodies and may affect the spread of antigen determinants [92]. Furthermore, they can affect several immune-related functions involved in thrombosis and autoimmune development [93].

One of the most unique facets of the defense arsenal that neutrophils carry is their ability to form NETs. NETs are strands of nucleic acids bound to nuclear, cytoplasmic, and granule proteins that are extruded into the extracellular space. NETs form in response to microbial and non-microbial danger signals such as platelets, immunocomplexes, autoantibodies, cytokines, drugs, and crystals (urate or calcium pyrophosphate in gout and pseudogout, respectively; cholesterol crystals in the atherosclerotic arterial wall) [5].

Several studies demonstrated NETs formation in circulating and synovial RA neutrophils, pointing at a convincing relationship between free circulating DNA levels and inflammatory markers such as CRP, erythrocyte sedimentation rate (ERS), and IL-17, together with ACPAs titers. In addition, it was found that the impairment in the NETs degradation in RA patients is mainly due to decreased DNase activity [94].

The formation of NETs can hasten RA development. The release of NETs promotes the production of ACPAs, favoring the production of inflammatory cytokines (such as IL-6 and IL-8), chemokines and adhesion proteins. ACPAs also induce the release of peptidyl-arginine deiminases (PADs, main inductors of NET formation) from neutrophils, which catalyze the change of arginine to citrulline, thus generating a continuous cycle of autoantibody production. Moreover, NETs contain layers of PADs that accumulate in the synovial fluid, thereby supporting the formation of citrullinated products [95,96].

Since plasma levels of cell-free nucleosomes has prominent specificity (92%) and sensitivity (91%) for the diagnosis of RA, NETs were proposed as a potential novel biomarker [31] and for “early arthritis” diagnosis [97].

The inflammatory milieu of the RA synovium appears particularly favorable to allow neutrophils to form NETs [98]. Thus, synovial RA neutrophils are primed to undergo spontaneous NET formation. Interestingly, RA autoantibodies (ACPAs and RF) and various proinflammatory cytokines can induce neutrophils to extrude NETs [94]. NETs within the synovial joint engage in putative pathogenic interactions with resident cells. Synovial NETs can be internalized by resident RA FLS in a receptor for advanced glycation end products (RAGE)–TLR9-dependent axis [92]. This internalization of NETs by FLS induces them to synthesize enhanced levels of proinflammatory cytokines and chemokines and endows them with antigen-presenting cell capabilities through MHC Class II (MHCII) induction [99]. Indeed, FLS that internalize NETs carrying citrullinated peptides can process and present these peptides to human RA antigen-specific T cells and activate them. Taken together, the interaction between synovial NETs and FLS may contribute to the activation of pathogenic innate and adaptive immune pathways in RA that could ultimately promote immune dysregulation, amplification of inflammatory responses, and cartilage damage.

Within the lungs, a site associated with initial stages of loss of self-tolerance in RA, increased NET complexes are found in at-risk first-degree relatives of RA patients [100]. These complexes correlate with mucosal IgA and IgG ACPAs generation, suggesting that local NET formation in the airways of individuals at risk of RA may be linked to the generation of autoantibodies in the preclinical state.

Periodontal disease also linked with RA development, is associated with a profound gingival neutrophilic infiltrative response, along with local enhanced NET formation [101]. It is possible that enhanced NET formation in these tissues is an early event in RA where PADs are activated and promote generation and externalization of citrullinated autoantigens.

Recent evidence suggests that NETs also contribute to the development of cardiovascular diseases, as they can promote tissue damage, inflammation, thrombosis, and atherosclerosis.

NETs were identified in atherosclerotic lesions from both mice and humans [102]. In addition, some proteins embedded in NETs (such as cathelicidins), can recruit monocytes into arteries and stimulate the dendritic cells in plaque lesions, thus promoting the development and aggravation of the atherosclerotic process [103]. Moreover, a recent study showed that hypercholesterolemia, one of the main factors provoking atherosclerosis, can produce NETs in vitro [104].

In the scenery of RA, we recently conducted a study to evaluate the relationship between NETosis and NETosis-derived products and the development of atherosclerosis and CVD in RA [31]. We firstly confirmed that NETosis is augmented in RA neutrophils, and that elements related to NETs extrusion were significantly enhanced in RA patients. In addition, we expanded these observations by examining the relationship between NETosis-derived products (such as cell-free DNA), and clinical and autoimmune parameters related to inflammation and CVD in RA patients, including inflammatory mediators, and oxidative stress markers. These results identified NETs as critical players in both the activity of the disease and the physiopathology of athero-thrombosis in this autoimmune condition.

Overall, these observations indicate that accelerated NETosis present in RA is directly involved on its pathogenesis, through the production of citrullinated autoantigens and immunostimulatory molecules that supports abnormal adaptive and innate immune responses in the joints and in the periphery thus inducing pathogenic mechanisms and the development of comorbidities in this disease.

### 4.2. Regulation of NETosis by Biological Therapies

Both conventional and biologic DMARDs were shown to critically modify neutrophil function. A recent review by our group [17] described in depth the mechanisms by which standard RA therapy can impact the inflammatory capacity of neutrophils. Indeed, antimalarials, glucocorticoids, leflunomide, methotrexate, anti-TNF agents, anti-IL-6receptor antibodies, and tofacitinib (a JAK-inhibitor) were reported to have some effect on neutrophil functionality.

Inflammatory cytokines, (i.e., TNFα, IL17a, IL-6 and IL-8) induce extrusion of NETs in RA neutrophils. In turn, NETs stimulate more production of cytokines and chronic inflammation by activating macrophages and/or FLS [94,105]. Therefore, NETs provoke inflammatory responses in RA FLS by stimulating the secretion of chemokines, cytokines, and molecules of adhesion. Besides, IL6 may promote B cell differentiation into plasma cells, which can produce ACPA, thus promoting indirectly more NET formation.

In this sense, it was shown that anti-TNF and anti-IL-6 receptor therapy can reduce neutrophil adhesion molecules and down-regulate the production of key neutrophil chemokines [37]. Tofacitinib prevents migration of neutrophils toward its key chemokine, IL-8 [45] and a recent study showed that it can decrease NET formation when administered in vivo to mice [106].

Accordingly, Dr Kaplan’ group demonstrated some years ago that the neutralizing antibodies to IL-17R can reduce RA serum-induced-NETs [94]. Thereafter, we reported the in vivo reduction in neutrophil elastase (NE) and myeloperoxidase (MPO) in neutrophils from RA patients after six months of treatment with the IL-6R inhibitor tocilizumab [39].

Therefore, neutrophils from RA patients treated with tocilizumab for six months showed reduced generation of NETs, so the area of DNA fibers stained with SYTOX was reduced by tocilizumab effects in RA neutrophils treated with phorbol myristate acetate (PMA). Moreover, a diminished release of cell-free nucleosomes was observed in serum from RA patients after tocilizumab therapy, accompanying the reduction of NETosis generation by tocilizumab observed at cellular level.

To evaluate the specificity of tocilizumab effects, we developed in vitro studies. Pretreatment of neutrophils isolated from RA patients with IL-6 for 6 h, promoted a significant increase in intracellular levels of NE and MPO that was prevented by adding tocilizumab. We further analyzed whether tocilizumab could diminish NETosis induced in vitro. The treatment of RA neutrophils with IL-6 or PMA induced an increase of NETosis formation that was prevented by the combined treatment of tocilizumab plus IL-6 or PMA generated, suggesting that tocilizumab might prevent NETosis in RA patients.

Later on, we conducted a new study to evaluate the relationship between NETosis and NETosis-derived products and the development of atherosclerosis and CVD in RA and the effects of anti-TNFα (i.e., infliximab) and other biologic drugs (such as tocilizumab) [31].

This study demonstrated, firstly that NETosis is increased in RA neutrophils, and that other molecules related to the extrusion of NETs are enhanced in RA patients. In addition, we extended these observations by examining the relationship among NETosis-derived products, such as cell-free DNA and clinical and autoimmune parameters, inflammatory mediators, and oxidative stress markers related to CVD in RA, thus identifying NETs as relevant players in both the activity of the disease and the physiopathology of atherothrombosis in this autoimmune condition.

In addition, we confirmed that bDMARDs, such as infliximab and tocilizumab, inhibit NETosis in parallel to the decline of both disease activity and expression of crucial inflammatory mediators. In vitro, inhibiting the secretion of NETs by DNAase, tocilizumab or infliximab can reduce endothelial dysfunction and the activation of circulating immune cells, thereby affecting the overall activity of the vascular system.

Recently, we proved [90] that rituximab treatment for three months of RA patients induced an early recovery of immune and vascular system states in parallel with a reduction in disease activity, and a quick restoration of the immune and vascular systems, comprising a re-setting in the circulating inflammatory and oxidative stress mediators levels, in conjunction with a significant reduction in the levels of NETosis-derived products. Furthermore, incubation, in vitro, of neutrophils from healthy volunteers with RA patients’ serum before and after rituximab therapy for three months demonstrated, firstly, an induction of NETosis in neutrophils, in addition to an increase in the levels of NETosis-derived products released to the cell culture medium, including elastase and DNA. Then, the NETotic process and the derived products released were reduced when neutrophils were treated with RA serum after rituximab therapy, in parallel with the reduced expression of inflammatory mediators by these cells.

Overall, NETosis-derived products might constitute a potential biomarker of diagnostic for disease activity status and early atherosclerosis development, as well as for the valuation of the therapeutic effectiveness in RA patients. All presented results can be helpful in the consideration of the medical application of analyzed drugs in RA patients, where the impact of NETosis processes may influence the progression of the disease, its comorbidities, and their therapeutic response.

## 5. Gene Expression

### 5.1. Gene Expression Signatures and Networks Associated with the Pathogenesis of RA

The study of the transcriptome through high-throughput technologies in bulk tissues and purified cell types from RA patients allowed the identification of new genes, pathways and networks associated with the pathogenesis of the disease.

Lewis et al. reported in a recent study the molecular networks that drive early RA progression through an extensive RNA-seq analysis in synovium and matched whole blood samples from 90 RA early treatment-naïve patients [107]. The analysis of synovial biopsies revealed that cell-specific genes modules, associated with ectopic lymphoid and myeloid responses, correlated with the disease activity and radiographic damage, suggesting that the infiltration of those immune cells influence the severity of RA from early on in the course of the disease. Comparison of synovial and whole blood samples exhibited large differences where the transcriptomic alterations seemed to be more drastic in synovial tissue. Synovial RNA-Seq gene clusters analysis identified enriched molecular networks associated with immune cell activation and maturation, pro-inflammatory intracellular signaling (phospholipase C, PI3K, and NFAT) and pro-fibroblast Wnt/ß-catenin pathways and correlated with distinct histological pathotypes. Ingenuity pathway analysis (IPA) also showed potential upstream regulators including IFNγ, IFN-α2, IFN-β1, IL-7, IL-21, and CD40L for biopsies with a predominant B-lymphocyte infiltration and TNFα, IFNγ, IL-1β, IL-4, IL-6 and IL-15 for biopsies with a predominant myeloid infiltration. Gene expression was associated with bone erosion at 12 months where synovial plasma cell genes at baseline were predictors of worse prognosis. Moreover, several synovial gene modules correlated with clinical response to conventional DMARDs at 6 months including modules related to IFN signature, monocyte and chemokine, dendritic cell, B cell and antigen presentation. These results were not observed in blood sample where no module was associated with clinical response.

A differential gene expression profile was also noticed between ACPA-positive and ACPA-negative patients, highlighting the role of these autoantibodies in the altered transcriptomic profile. In line with this, we also demonstrated in vitro that the incubation of healthy leukocytes with ACPAs purified from RA patients promoted a distinctive alteration of the expression of pro-inflammatory genes [10]. These autoantibodies increased the expression of IL-1β, CCL2, IL8, VEGF in monocytes and neutrophils, TNFα in monocytes, IL6 in lymphocytes and monocytes, IL2 in lymphocytes and reduced the expression of IL10 in lymphocytes. Gertel S et al. validated these results in peripheral blood mononuclear cells (PBMCs) showing that ACPAs bind specifically immune cells through the Fab portion [108].

Along with autoantibodies characteristic of the disease, other key factors of the pathogenesis of RA, such as TNFα, can induce a differential gene expression in specific cell types. Loh C. et al. described that the treatment of macrophages and fibroblast-like synoviocytes (FLS) from RA patients with TNFα induced a set of 280 genes whose expression is transient in macrophages (peak at 6 h) but sustained in FLS (peak at 24–72 h) [109]. The molecular functional analysis revealed that these genes were associated with inflammatory pathways implicated in RA and had upstream regulators such as TNFα and NF-κB. Moreover, a large number of genes which are repressed in macrophages after a second stimulation are triggered again in FLS. These data suggested that a fraction of inflammatory genes induced by TNFα is not effectively terminated in FLS and can contribute to maintain the inflammatory status of RA patients. FLS also showed a distinctive expression profile depending on the location. Ai R. et al., showed by RNAseq analysis that the gene expression profile of FLS was divergent between knee and hip where 107 genes were differentially expressed associated with pathways such as acute phase response signaling, IL-6 signaling or protein citrullination among others [7].

The altered gene expression profile of RA patients compared with healthy donors and related diseases was characterized in different cell types from peripheral blood and synovium including PBMCs, neutrophils, macrophages, or dendritic cells. Wright H.L. et al. performed RNA-seq in purified neutrophils from HDs and RA patients and 889 genes were differentially expressed including 666 genes up-regulated and 223 genes down-regulated in RA compared with HDs [110]. The functional analysis of the deregulated genes by using IPA analysis revealed that 178 genes were predicted to be regulated by IFNα, β, and γ. Moreover, cluster analysis identified two subgroups of RA patients according to the response to IFN which were designated as IFN low and IFN high. The expression of the genes of each cluster was correlated with the response to Anti-TNFα at 12 weeks, where patients from the IFN high group responded better to this therapy. Choi S. et al. performed a microarray analysis of mRNA expression in macrophages purified from synovial fluid of RA patients and macrophages differentiated from peripheral CD14^+^ monocytes [111]. This analysis showed that 1913 genes were differentially expressed in RA macrophages compared with HDs, where 1015 genes were up-regulated and 898 genes were down-regulated. The cellular processes enriched by these genes were related to leukocyte activation, chemotaxis, cytokine production, apoptosis, and cell death among others. Besides, they performed an analysis to investigate potential transcription factors responsible for the differential expression of those genes including NFAT5, SPI1, SP1, NF-κB complexes, TP53, PPARγ, and E2F4. Due to the central role of dendritic cells (DCs) in all immune responses, Cooles F. et al. evaluated the transcriptomic profile of CD141^+^ and CD1c+ conventional DCs (cDCs) and plasmacytoid DCs (pDCs) in 44 early, drug-naïve RA patients and 30 healthy donors by using a Nanostring panel of 584 genes related to immunology [6]. Several genes associated with proliferation and inflammatory cytokine signaling were up-regulated in RA pDCs (IFNAR1, IL6R, CSF1R, MAPK14, IL6ST) and RA CD1c+ DCs (BCL3, TNFα, CCR6, ICOSLG, BTLA, IKZF1, IL6R, IRF8) while apoptotic genes were down-regulated (CASP3 and CASP8). Furthermore, pDCs had similar levels of IFN-I and IFN-III mRNA transcripts as other leukocyte subsets such as CD4+, and CD8+ T cells, B cells, and monocytes.

Along with the differences found between RA patients and healthy donors (HDs) in multiple cell types, it was also shown that RA patients have a distinctive gene expression signature in relation to other immune-mediated diseases. Thus, Petrackova A. et al. analyzed in PBMCs a gene signature of the innate immune system including Toll-like receptors (TLRs) and key members of the interleukin (IL)1/IL1R and CXCL8/IL8 families in 36 active RA patients, 28 active systemic lupus erythematosus (SLE) patients and 22 active systemic sclerosis (SSc) patients [112]. RA patients showed the up-regulation of several genes such as TLR5, TLR2, and SIGIRR/IL1R8 compared with SLE and TLR5, TLR3, IL1RAP/IL1R3, SIGIRR/IL1R8 and IL18R1 in relation to SSC. Furthermore, RA patients exhibited an increased expression of TLR8, TLR5, TLR3, TLR2, IL18, IL18R1, IL1B, IL1RN, IL1RAP, and SIGIRR/IL1R8 compared with HDs.

### 5.2. Modulation of the Transcriptomic Profile of RA Patients by Effect of Biological Therapies

The modulation of the gene expression in peripheral blood cells and synovium of RA patients by using biological therapies was reported and linked to their therapeutic efficacy in the control of the disease activity.

Pachot A. et al. studied by RT-PCR the early and late changes of TNFα mRNA in whole blood of 44 RA patients promoted by Infliximab infusion [113]. First, the expression level of TNFα before therapy was significantly increased compared to 27 HDs and was similar between responder and non-responder patients. After 4 h of infliximab infusion the mRNA expression of TNFα was significantly reduced in RA patients. Moreover, after 22 weeks this reduction was even more prominent. No differences in gene expression changes were observed between responder and non-responder patients.

Several studies analyzed by high-throughput technologies changes in the whole transcriptome of RA patients in whole blood and PBMCs treated with Anti-TNFα therapies. Sekiguchi N. et al. evaluated changes in gene expression promoted by infliximab in peripheral blood from 18 RA patients by Agilent Microarray platform at 2, 14 and 22 weeks [114]. Approximately 15% of the total genes were differentially expressed comparing the baseline and one or more points evaluated during the 22 weeks of treatment. Differentially expressed genes correlated with the disease activity and inflammatory markers such as CRP levels, highlighting the role of IFN-related genes as key players in those processes. Responder patients following American College Rheumatology (ACR) criteria reduced the increased expression of inflammatory genes at 2 weeks and remained at normal levels during the time evaluated. Non-responder patients decreased the expression levels of inflammatory genes at 2 weeks but returned to the baseline after 14 weeks. Responder and non-responder patients also showed differential expression of some genes at baseline such as CX3CR1, IL2RB and chemokine ligand four genes as well as TNF-related genes. Meugnier E. et al. also identified 251 genes differentially expressed (178 up-regulated and 73 down-regulated) in PBMCs of responder RA patients after 12 weeks of treatment with either adalimumab (ADA) or etanercept (ETN) [115]. The functional analysis of the genes modulated by anti-TNFα therapy was related to immune response, apoptosis, inflammation, mitochondrial oxido-reduction, and protein synthesis.

Lequerré T. et al. showed the potential of gene expression as predictor of therapy response at 3 months in 33 RA patients treated with infliximab by microarray technology [116]. A signature of 20 genes related to immune response in PBMCs identified the group of responder patients following European League Against Rheumatism (EULAR) criteria, with 70% of specificity and 90% of sensitivity. The gene signature was also validated by RT-PCR in a validation cohort. The expression of this gene signature did not change after anti-TNFα therapy in responder patients while the expression of most of those genes was reduced after treatment in non-responder patients. In line with this, Helen L. et al. also showed that the expression of a signature of three genes from neutrophils of RA patients including CMPK2, IFIT1B and RNASE3 at baseline was predictor of anti-TNFα response following EULAR criteria at 12 weeks with an area under the curve of 0.94 [117]. The results obtained by RNA-seq were validated in two independent cohorts of RA patients by RT-PCR. Tasaki S. et al. compared the different response of infliximab, tocilizumab and methotrexate at transcriptional level [118] in whole blood from 45 RA patients at 24 weeks. Globally, infliximab and tocilizumab normalized the transcriptional alterations of RA patients to HDs levels more efficiently than methotrexate. These changes mainly occurred in genes that were expressed at high or low levels in neutrophils suggesting that neutrophil gene expression was modulated by these therapies. Moreover, TCZ normalized gene signatures that could not be modulated by infliximab and methotrexate, suggesting a more potent effect of this drug at transcriptional level. We also demonstrated in vitro that the treatment of neutrophils from RA patients with tocilizumab and infliximab normalized the levels of key inflammatory mediators including VEGF-A, TNFα, IL1-ß, IL8, IL6R and STAT3 [32].

The transcriptomic changes promoted by biological therapies were also reported with other drugs such as Rituximab (RTX). Thus, Sellam J. et al. analyzed changes in gene expression of PBMC from 68 RA patients before and after 24 weeks of rituximab therapy by microarray technology [119]. A total of 339 genes were differentially expressed at 24 weeks compared with baseline with no differences between responder and non-responder patients. The most important changes were those associated with the down-regulation of genes linked to the B cell development and primary immunodeficiency signaling (CD20, CD22, BLNK, BANK, Pax5, Immunoglobulins, TLR10, SP1B, and FCER2). These results were further validated by RT-PCR confirming the down-regulation of those genes after RTX. Furthermore, the expression of a signature of 143 genes characterized by the up-regulation of inflammatory genes such as NFKB, IL33 and STAT5 and the down-regulation of the interferon pathway were predictors of therapy response to rituximab with high accuracy. Similarly, Vanessa EH. et al. analyzed a selected panel of 125 genes related to RA pathogenesis in synovial arthroscopy samples from 20 RA patients before and after 3 and 21 months of rituximab therapy [120]. After three months TNFSF11 was up-regulated and IgM, LTβ and S100A12 was significantly reduced. Besides, after 21 months multiple genes were down-regulated including genes involved in T and B cell biology (FOXP3, CD27, CD8, EOMES, CD38, CD52, LCK, GRZA, CTLA4, CD122), cytokines, chemokines, receptors, several interleukins (IL6, IL13, IL12, IL17RA, IL23a). In parallel, the synovial expression of genes related to T cell, macrophage, remodeling and interferon-α biology at baseline is associated with the magnitude of early and late response to rituximab.

Another biological therapy, abatacept, also showed its capacity to modulate gene expression. Sumitomo S. et al. recently analyzed by RNA-seq the expression profile of seven CD4 T cell subsets (Th1, Th17, Th1/17, nonTh1/17, Tfh, Treg and naive) from RA patients before and after 6 months of abatacept [121]. This treatment promoted a large shift toward the expression profile of HDs. Most of the up-regulated and down-regulated genes at baseline were restored after abatacept therapy. Weighted gene co-expression network analysis (WGCNA) identified a module of highly connected genes associated with the dysregulation of the TCR signaling pathway correlated with the disease activity (DAS28-CRP) which was ameliorated by the therapy.

Thus, the analysis of the transcriptome in different cell types and tissues from RA patients is an important source of information to better understand the complex mechanisms underlying RA pathogenesis and the therapeutic response to biological drugs.

## 6. MicroRNAs

### 6.1. Role of MicroRNAs in the Pathogenesis of RA

MiRNAs are short non-coding RNAs of 18–22 nucleotides that regulate gene expression at the post-transcriptional level. miRNAs bind to the 3′UTR region of messenger RNAs (mRNAs) of its target genes by complementary in sequences and inhibit the translation into proteins or promote mRNA degradation through mRNA deadenylation and decapping [122].

The biogenesis of miRNAs begins with the transcription of a long hairpin primary miRNA (pri-miRNA) by the RNA polymerase II/III which is then cleaved by the RNase III enzyme Drosha along with the RNA binding protein DiGeorge Syndrome Critical Region 8 (DGCR8) generating a pre-miRNA. Exportin 5 (XPO5)/RanGTP complex allows the exportation of the pre-miRNA into the cytoplasm, where another RNase III enzyme, Dicer, cleaves and generate an intermediate miRNA duplex. One strand of the mature miRNA is finally loaded in the RNA-induced silencing complex (RISC) that facilitate the mRNA target recognition. This complex is integrated by the mature miRNA, Ago proteins, Dicer and a trans-activation-responsive RNA- binding protein (TRBP) [123]. The miRNA biogenesis process was shown to be impaired in different pathological contexts. Our group recently reported that neutrophils from peripheral blood of RA patients exhibit a global down-regulation of the miRNA profile associated with clinical features, which was even more prominent in neutrophils from synovial fluid [32]. This fact was in line with the down-regulation of different biogenesis machinery proteins such as Dicer, Ago-1, Ago-2 and Xpo-5 in RA neutrophils compared with HDs. This impaired miRNA biogenesis process in neutrophils was also confirmed by our group in other autoimmune disease such as antiphospholipid syndrome and systemic lupus erythematosus [124].

miRNAs not only play a central role in controlling biological processes, such as development, differentiation, apoptosis and survival, but also the underlying mechanisms of the innate and adaptive immune response [125]. MiRNAs seem to have an altered expression in several cells, biofluids and tissues in patients with autoimmune disorders [126]. Aberrant miRNA expression in RA was associated with enhanced inflammatory signaling pathway, increased secretion of pro-inflammatory cytokine and chemokines and activation of several immune cell types [127]. Moreover, altered expression of circulating miRNAs in whole blood, serum, plasma, urine, and synovial fluid were identified in RA patients and associated with the disease activity, prognosis and therapy response, highlighting its role as biomarkers of disease [8].

Despite several studies characterized specific and distinctive altered miRNA profiles of RA patients in different types of samples, there are several miRNAs that seem to share a key role in the pathogenesis of RA, such as miR-146a, miR-155, miR-221, miR-222 and miR-223.

miR-155-5p is a central regulator of B cell function and development and T cell response and functions, which control the expression of cytokines such as IL-1 and TNFα. MiR-155 is increased in the sera of RA patients [128], as well as in synovial macrophages where promote cytokine production and survival [129]. The increased levels of this miR were also correlated with high DAS28 [130]. Moreover, the genetic silencing of miR-155 gene by CRISPR/CAS-9 led to resistance to arthritis development through the inhibition of proinflammatory cytokines production [131].

miR-146a-5p is a negative regulator of NF-kB activation, which is increased in many cell types in RA patients such as CD4^+^ T cells, and Th17 cells, peripheral blood mononuclear cells, and synovial fibroblasts. Niimoto T. et al. also demonstrated the association between the expression of miR-146a and IL-17 in synovium and PBMCs of RA patients [132]. Besides, Li. et al. showed that the expression of miR-146 in CD4+ T cells from peripheral blood and synovium of RA patients increased and positively correlated with levels of TNFα [133].

The expression of miR-221 and miR-222 in PBMCs from RA patients were directly associated with the increased production of inflammatory mediators [134], disease activity [135] and osteoblastogenesis [133]. In the same line, increased levels of miR-223-3p in synovial macrophages of RA patients were linked to the enhanced secretion of pro-inflammatory cytokines [136,137].

### 6.2. MicroRNAs as Biomarkers of Therapy Response and Modulation by Biological Therapies

The effects of biological therapies in the normalization of the altered miRNA profile in RA and the levels of its predicted inflammatory targets were demonstrated. Furthermore, the basal level of circulating miRNAs was associated with the therapy response highlighting their potential role of biomarker predictors of clinical efficacy.

We evaluated the role of anti-TNFα therapy in the regulation of the expression of miRNAs in the serum of 95 RA patients [36]. Through a PCR-Array that included a selected panel of 84 circulating miRNAs associated with different diseases, we analyzed the changes produced by Anti-TNFα therapy (etanercept, infliximab and adalimumab) before and after 24 weeks. The expression levels of 75 and 9 miRNAs increased and reduced respectively after the therapy in an exploratory cohort. To validate the array data, 10 miRNAs with a fold change higher than 2 and involved in processes such as inflammation and immune response were selected including miR-125b, -23a-3p, -21-5p, -126-3p, -146a-5p, let-7a-5p, -16-5p, -124a-3p, -155-5p, -223-3p. Six miRNAs were validated by RT-PCR in the whole cohort of RA patients where miR-125b, -126-3p, -146a-5p, -16-5p, -23-3p, and miR-223-3p were significantly increased after 6 months of Anti-TNFα therapy. The analysis of the changes between responder and non-responder patients following EULAR criteria revealed that in responder patients the expression level of those miRNAs was strongly increased after the therapy while in non-responder patients remained without change. Moreover, a parallel reduction of rheumatoid factor and pro-inflammatory cytokines such as TNFα, IL-6 or IL-17 was observed in responder patients. Correlations between the changes of these miRNAs and changes in DAS28 and CRP levels were also identified. The IPA functional analysis of the predicted targets of this miRNA signature suggested that they were involved in pathways directly associated with the disease such as the role of IL-17A in RA, the role of osteoblasts, osteoclasts and chondrocytes in RA, the role of macrophages, fibroblasts and endothelial cells in RA, and STAT-3 or IL-6 signaling. The potential role of these circulating miRNAs as biomarkers predictors of therapy response was also analyzed, and the expression level of miR-23a and miR-223 before therapy identified non-responder patients with high accuracy. The combination of both miRNAs at baseline in a statistical model improved the prediction of the Anti-TNFα therapy response at 6 months.

In a recent study, we also analyzed the effects of other biological therapy, such as rituximab, in the altered circulating miRNA profile of RA patients [90]. The analysis of the miRNome in the serum of RA and HDs through next-generation sequencing revealed an altered expression of 198 miRNAs in an exploratory cohort. The altered expression of four miRNAs, selected based on its role in the pathogenesis of RA after a functional analysis of predicted targets, was validated by RT-PCR in an independent cohort. The effects of RTX therapy was studied before and after 3 months of treatment in 16 RA patients and the normalization of the altered expression of these miRNAs was noticed. RTX therapy promoted the up-regulation of miR-146a-3p and the down-regulation of miR-16-5p, -125a-5p and -23a-3p, which were in line with the restoration of circulating molecules related to oxidative stress, NETosis and inflammation and the subsequent clinical response.

We also tested in vitro the effect of biological therapies such as infliximab and tocilizumab in neutrophils from RA and HDs [32]. Firstly, we evaluated the treatment of neutrophils from HDs with TNFα and IL6 on the expression of a selected panel of miRNAs related with biological pathways associated with the disease. Both cytokines promoted the down-regulation of most of the analyzed miRNAs where TNFα exhibited a stronger effect. Secondly, the treatment of RA neutrophils with infliximab and tocilizumab induced the up-regulation of these miRNAs being the effect of IFX more accused.

Other studies also reported the modulation of key miRNAs involved in the pathogenesis of RA with Anti-TNFα therapy. Cheng P. et al. analyzed the changes promoted in the expression of miR-125a and -125b after infliximab therapy in the serum of 96 RA patients. Both miRNAs were highly up-regulated in RA patients compared with HDs and correlated with DAS28, ESR and CRP levels [138]. After infliximab treatment the levels of these miRNAs were gradually reduced at week 4, 12 and 24 which was in line with the gradually increase of the clinical response rate. Moreover, the level of miR-125b before therapy was higher in responder RA patients in relation to non-responder RA patients. The combination of clinical data such as disease duration, CRP levels and history of biologics along with miR-125b in a combined panel predicted the therapy response with high accuracy (ROC: 0.82). Similarly, Duroux-Richard I. et al. showed that high circulating levels of miR-125b before therapy was predictor of response to rituximab after 3 months of therapy in 16 responders and 16 non-responder patients by RT-PCR [139]. Sode J. et al. also reported the down-regulation of the plasmatic levels of miR-27a-3p by effect of ADA after three months in 89 RA patients [140]. Besides non-responder RA patients did not change the levels of miR-27a-3p. A multivariate model integrated by the expression of ten miRNAs at baseline including miR-27a-3p, -27b-3p, -146b-5p, -19b-3p, -16-5p, -423-5p, -23a-3p, -106a-5p, -29b-3p and -17-5p predicted the clinical response to ADA with an AUC of 0.82. Ciechomska M. et al. also showed the down-regulation of miR-5196 after 6 months of Anti-TNFα therapy in RA and Ankylosing Spondylitis patients. This miRNA was found previously up-regulated in both group of patients compared with HDs [141]. Moreover, changes in the level of miR-5196 also correlated with changes of DAS28 and CRP levels.

Other studies exclusively aimed to identify miRNAs as biomarkers predictors of response to Anti-TNFα using miRNAs in different sources such as whole blood, serum and PBMCs. Krintel SB. et al. evaluated in whole blood samples from 89 early naïve treated RA patients 337 miRNAs by using TaqMan Human MicroRNA assay [142]. The combination of low levels of miR-22 and high levels of miR-886 before therapy predicted the good response following EULAR criteria after 12 months with high accuracy. Cuppen B. et al. studied the profile of 758 miRNAs in serum samples from 80 patients before anti-TNFα therapy including 40 adalimumab and 40 etanercept [143]. High levels of miR-23a and miR-197 at baseline predicted good response to etanercept after 1 year, while low and high baseline levels of miR-143 and miR-99 predicted good response to ADA. The addition of the miRNA information to predictive models integrated by clinical variables such as DAS28, SJC, CRP and other treatments increased the AUC-ROC of the models from 0.75 to 0.97 for ADA and from 0.68 to 78 with etanercept. Despite these promising results, they could not validate the data in an independent cohort of 40 patients highlighting the need for a minimum of standards in experimental and technical design in order to increase the reproducibility. Liu Y. et al. recently evaluated the potential of miRNAs in PBMCs from RA patients as biomarker predictors of therapy response to etanercept after 24 weeks [144]. A microarray analysis was performed in an exploratory cohort of 10 responder and non-responder patients and 137 miRNAs were differentially expressed and related to immune response and inflammation. The top-10 dysregulated miRNAs were then analyzed in 90 RA patients before ETN by RT-PCR including miR-146a-5p, -192-5p, let-7a-5p, -19b-3p, -320c, -335-5p, -149-3p, -766-3p, -24-3p and -1226-5p. Validation RT-PCR analysis confirmed the up-regulation of 146a-5p and the down-regulation of let-7a-5p in responder patients. A combined model integrated by these two miRNAs, CRP levels and biologics history presented a great predictive value for clinical response (AUC of 0.86). The potential of miRNAs as biomarkers of therapy response was also shown in patients treated with conventional DMARDs such as methotrexate where miR-132, miR-146, miR-155 and miR-10 were identified as good candidates [145,146].

Biological therapies seem to achieve the clinical response in part through the normalization of altered microRNAs with key functions through relevant targets associated with immune and inflammatory pathways present in RA. Moreover, the levels of miRNAs at baseline showed potential to be biomarkers predictors of therapy response in serum, whole blood and PBMCs. The replication of new studies with standard methodologies and bigger cohort of patients will be needed in the future to confirm or discard its utility to guide clinical decisions.

## 7. DNA Methylation

### 7.1. Contribution of DNA Methylation to the Pathogenesis of RA

DNA methylation is an epigenetic mechanism which involves the addition of a methyl group (CH3) to the C-5 position of the cytosine ring of DNA. This process is usually linked to the inhibition of the binding of transcription factors on the promotor region of gene resulting in the repression of the gene expression. The methylation process is mediated by specific enzymes such as DNA methyltransferase (DNMT), DNMT1, DNMT3A and DNMT3B in response to environmental stimuli, other stress or during development. In parallel other enzymes are responsible for the demethylation of the DNA including ten-eleven translocation enzymes (TET), TET1, TET2 and TET3 [147].

Recent studies revealed the key role of methylation in the pathogenesis of RA. Genome-wide methylation studies reported a full spectrum of changes in FLS and immune cells during the onset and progression of the disease.

FLS have a unique methylome with a specific pattern linked to the joint localization and disease state. Nakano et al. demonstrated that FLS from RA patients exhibited a global DNA hypomethylation associated with an increase expression of genes related to extracellular matrix proteins, matrix degrading enzymes, adhesion molecules, growth factor etc. This was in line with the reduction of the protein level of DNMT1 in FLS from RA patients which was also down-regulated in vitro by cytokines and growth factors stimulation [148,149]. Karouzakis E. et al. compared the whole methylome of synovial fibroblast from patients with early RA, resolving RA, fully established RA and from non-RA patients by using the Infinium Human methylation arrays [150]. A significant number of CpG sites in the promotor region of important genes associated with the pathogenesis of RA were differentially methylated among the different disease stages suggesting that the DNA methylation patterns are not random and occur during the development and progression of the disease.

Concerning immune cells, recent studies identified a global DNA hypomethylation in monocytes and T Cells from RA patients compared to HDs along with a lower expression of DNA methyltransferase 1 (DNMT1) [151]. In T cells and B cells from 23 early RA naïve treated patients, specific aberrant methylation profiles were also characterized by microarrays platforms compared with HDs supporting the role of methylation changes in the development of RA [152]. A clear example that aberrant DNA methylation can affect immune cell functions was published by Cribbs A. et al. [153]. They observed in T cells from RA patients that the hypermethylation of the promoter region of CTLA-4 prevented the binding of the nuclear factor of activated T cells (NF-AT) which decreased CTLA-4 expression resulting in a failure to activate the immunomodulatory kynurenine pathway of Treg cells. Besides the treatment with MTX induced hypomethylation of FoxP3 increasing the gene expression of FoxP3 and CTLA4 which normalized Treg function in RA patients [154]. The interaction between DNA methylation and gene expression needs of multilayer analysis through integrated analysis. Zhu. et al. recently performed an integrated analysis of DNA methylation and gene expression in PBMCs from RA patients where identified an interferon inducible gene interaction network (MX1, IFI44L, DTX3L and PARP9) and highlighted the key role of PARP9 gene in the pathogenesis of RA [155].

Since methylation is a reversible process, the alteration of the DNA methylation status in key genes and cell types could therefore be considered to be therapeutic targets as well as potential biomarkers for diagnosis and prognosis of RA patients.

### 7.2. Role of DNA Methylation in the Therapy Response of RA Patients

The methylation status of RA patients was shown to be modulated by conventional anti-rheumatic drugs in different immune cell types and linked to the therapy response of biological therapies.

De Andres, M. C et al., analyzed DNA methylation and the expression of seven methylation-specific enzymes in T, B, NK cells, monocytes and PBMCs from 19 early RA patients before and after 1 month of MTX and 17 HDs [151]. The low global methylation profile of RA leukocytes increased after MTX in three cell types including T cells, B cells and monocytes while no changes were detected in the rest of populations. Furthermore, the low expression level of the key methyltransferase DNMT1 increased after MTX in T cells, B cells and monocytes. This study validated the previous observations of Kim Y. et al. in smaller cohorts of RA and PsA patients where the methylation status was also reverted by long-term treatment with MTX [156]. These data highlight the interaction between DNA methylation and MTX that might contribute to the therapeutic efficacy of this drug in RA.

Plant D. et al. analyzed the methylome of whole blood from 72 RA patients before ETN therapy by using Infinium Methylation Assay from Illumina platform [157]. The methylation profile at baseline of responder and non-responder patients to anti-TNFα therapy was compared. Globally, non-responder patients showed higher methylation levels compared to good responders. The largest mean difference was 15% between both groups, where five differentially methylated positions was highly correlated with the response. Two of the top 5 CpG were located in the gene LRPAP1 (LDL Receptor Related Protein Associated Protein 1) being more methylated in non-responders. Single nucleotide polymorphisms of this particular gene were also associated with the therapy response. These results showed the potential role of DNA methylation of specific genes for predicting the therapy response to biological therapies.

Tao W. et al. recently analyzed the methylation profile of PBMC from 80 RA patients before two different Anti-TNFα therapies including adalimumab and etanercept and identified a distinct DNA methylation profile associated with the response [158]. Approximately 46% of the differentially methylated CpG positions (DMPs) were hypermethylated in ADA responders while 76% of DMPs were hypermethylated in ETN responders. Thus, at the epigenetic level it seems to exist a distinct mechanism defining the response to each biological drug in PBMC of RA patients. Besides when they applied machine learning methodologies to combine the DMPS for predicting the response to these drugs, different predictor models with an overall accuracy of 88% and 84.7% for etanercept and adalimumab, was identified respectively.

Overall, epigenetics mechanisms such as DNA methylation are emerging as new processes associated with the therapeutic response of RA patients as well as new potential biomarkers for predicting the therapy response to biological drugs through a personalized medicine approach.

NETs, neutrophils extracellular traps; MPO, myeloperoxidase; ROS, reactive oxygen species; LPO, lypoperoxides; N-Tyr, nitrotyrosine; MDA, malondialdehyde; SOD, superoxide dismutase; CAT, catalase; GPx, glutathione peroxidase; GSH, glutathione, miRNAs, microRNAs.

## 8. Conclusions

As discussed in this review, the diversity of biological processes underlying the pathogenesis of RA suggests that their clinical phenotypes represent jointly altered pathways rather than exemplify the outcomes of single altered entities.

Thus, the clinical picture of joint involvement in RA is the result of chronic inflammation of the synovium, characterized by multifaceted interactions of resident cells such as FLS with cells of the innate (e.g., macrophages, dendritic cells, neutrophils) and adaptive immune system (e.g., B and T lymphocytes). Besides, it was proven that very few cytokines (if any) have exclusively proinflammatory or anti-inflammatory functions, but rather work in a highly context-dependent manner that varies between the disease processes and tissues involved.

Moreover, similar to known inflammatory and immune responses, other novel dysregulated networks, such as oxidative stress and NETosis were found to jointly contribute to the pathogenesis of RA. Finally, environmental stress, which can be reflected in the genome as converging altered epigenetic marks, also influences gene regulation, and contributes to disease mechanisms. Therefore, understanding the genetic and the epigenetic landscapes using unbiased methods can potentially identify nonobvious pathways and genes that are responsible for synovial and systemic inflammation as well as for organ damage and associated comorbidities.

The progress in the understanding of the pathophysiology of RA provided a rational basis for the development of biologic disease-modifying antirheumatic drugs (bDMARDs) which have innovative mechanisms of action, based on the inhibition of specific molecular or cellular targets directly involved in disease pathogenesis. Moreover, contemporary studies have uncovered multiple pathways and processes underlying the action of each specific bDMARD.

**Figure 3:**
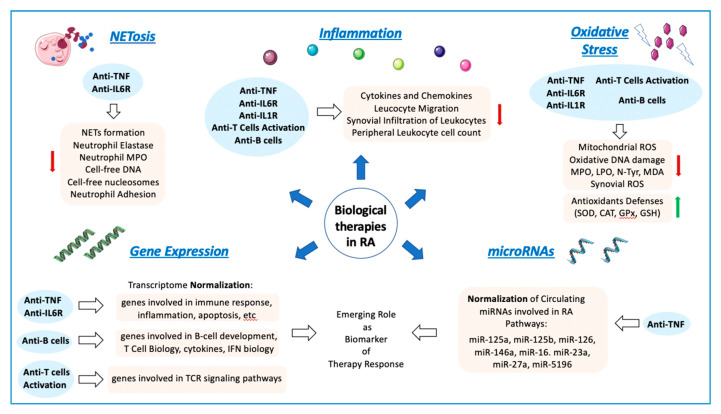
Molecular Effects of Biological therapies in RA patients. Biological therapies in RA have shown a wide range of beneficial molecular effects, by controlling numerous mechanisms altered in the disease. Hence, several therapies reduced the protein levels of circulating inflammatory mediators as well as the migration rate of activated immune cells to joints. Oxidative stress and NETosis have been also ameliorated after biological therapies including the reduction of ROS and oxidative markers, and the increase of the antioxidant defenses as well as the decrease of the NETs formation by neutrophils and related derived products. The altered transcriptome profile in peripheral blood and synovium of RA patients has been found normalized after biologics drugs, on which genes involved in key molecular pathways associated with the disease were modulated. Levels of microRNAs controlling the expression of central inflammatory genes have been found modulated in peripheral blood, plasma, and synovial fluid after anti-TNF therapy and some of, them along with mRNAs, are emerging as potential biomarkers predictors of therapy response.

Biological therapies demonstrated numerous beneficial molecular effects that modulate pathological processes of the disease (Figure 3). Thus, several bDMARDs down-regulated protein levels of circulating inflammatory mediators as well as the migration rate of activated immune cells to injured tissues. Pathologic oxidative stress and NETosis were also reversed, through the reduction of both ROS and the products of lipid and protein oxidative damage, along with the decrease in NETs formation by neutrophils and related derived products. Likewise, the altered transcriptome profiles found in peripheral blood and synovium of RA patients were found normalized after biologics, reversing the altered expression of genes involved in key molecular pathways associated with the disease. Accordingly, levels of microRNAs controlling the expression of central inflammatory genes were found to bemodified in peripheral blood, plasma, and synovial fluid after anti-TNF therapy and even some of them are emerging as potential biomarkers as predictors of therapy response.

Nevertheless, a significant percentage of RA patients do not display an adequate clinical response to these treatments. Therefore, based on all this accumulated knowledge, and in order to confer an optimal treatment program for RA patients, it would be necessary to settle the basis for the development of the so-called precision medicine approaches, which must necessarily involve a combined molecular and clinical characterization of each patient along with an integrated analysis of pathways related to non-responsiveness.

## Abbreviations

ACPAs	Anti-Citrullinated Protein Antibodies
ACR	American College of Rheumatology
APC	Antigen-Presenting cells
CAT	Catalase
CD	Cluster Differentiation
CRP	C Reactive Protein
CVD	Cardiovascular Disease
DAS	Disease Activity Score
DMARDs	Disease-Modifying Anti-Rheumatic Drugs
DMPs	Differentially Methylated CpG Positions
DNA	Deoxyribonucleic acid
DNMT	DNA Methyl Transferase
ERS	Erythrocyte sedimentation rate
EULAR	European League Against Rheumatism
FLS	Fibroblast-like synovial cells
GM-CSF	Granulocyte-macrophage colony stimulating factor
GPx	Glutathione Peroxidase
GSH	Glutathione
IFNγ	Interferon gamma
IL	Interleukin
ILC	Innate Lymphoid Cells
IPA	Ingenuity Pathway Analysis
LPO	Lipoperoxides
MAPK	Mitogen Activated Protein Kinase
MCP-1	Macrophage Chemoattractant Protein 1
MPO	Myeloperoxidase
NE	Neutrophil Elastase
NET	Neutrophil Extracellular Trap
NF-kB	Nuclear Factor -kappa B
NO	Nitric Oxide
PAD	PeptidylArginine Deiminase
PBMCs	Peripheral Blood Mononuclear Cells
pDCs	Plasmacytoid dendritic cells
PMA	Phorbol 12-Myristate 13-acetate
PNAd	Peripheral Node Addressin
RA	Rheumatoid Arthritis
RAGE	Receptor for Advanced Glycation End-products
RF	Rheumatoid Factor
ROS	Reactive Oxigen Species
SLE	Systemic Lupus Erythematosus
SOD	Superoxide Dismutase
SSc	Systemic Sclerosis
STAT6	Signal Transducer and Activator of Transcription 6
TET	Ten-Eleven Translocations
TGFβ	Transforming Growth Factor beta
Th	T helper
TLR	Toll Like Receptor
TNFα	Tumor Necrosis Factor alpha
Treg	Regulatory T cells
VAP1	Vascular Adhesion Protein 1
VCAM1	Vascular Cell Adhesion Protein 1
